# A translation invariant bipolaron in the Holstein model and superconductivity

**DOI:** 10.1186/s40064-016-2975-x

**Published:** 2016-08-08

**Authors:** Victor Lakhno

**Affiliations:** Institute of Mathematical Problems of Biology, Keldysh Institute of Applied Mathematics, Russian Academy of Sciences, Pushchino, Moscow Region 142290 Russia

**Keywords:** Delocalized, Broken symmetry, Strong coupling, Canonical transformation, Hubbard Hamiltonian, Bose condensate

## Abstract

Large-radius translation invariant (TI) bipolarons are considered in a one-dimensional Holstein molecular chain. Criteria of their stability are obtained. The energy of a translation invariant bipolaron is shown to be lower than that of a bipolaron with broken symmetry. The results obtained are applied to the problem of superconductivity in 1D-systems. It is shown that TI-bipolaron mechanism of Bose-Einstein condensation can support superconductivity even for infinite chain.

## Background

The problem of possible existence of superconductivity in low-dimensional molecular systems has long been of interest to researchers (Williams et al. 1992; Ishiguro et al. 1998; Toyota et al. 2007; Inzelt 2008; Lebed 2008; Altmore and Chang 2013). Presently, it is believed that this phenomenon may occur via a bipolaron mechanism. In three-dimensional systems a bipolaron gas is thought to form a Bose condensate possessing superconducting properties. It is well known that in one-and two-dimensional systems the conditions for bipolarons formation are more favorable than in three-dimensional ones. The main problem in this regard is the fact that in one- and two-dimensional systems Bose-condensation is impossible (Ginzburg 1968).

In papers Tulub (1962), Lakhno (2010, 2012, 2013) and Kashirina et al. (2012) a concept of translation invariant polarons and bipolarons was introduced. Under certain conditions these quasiparticles can possess superconducting properties even if they do not form a Bose condensate. Papers Tulub (1962), Lakhno (2010, 2012, 2013) and Kashirina et al. (2012) dealt with three-dimensional translation-invariant polarons and bipolarons. In the context of the aforesaid it would be interesting to consider the conditions under which translation invariant bipolarons arise in low-dimensional systems. Here the results of Tulub (1962), Lakhno (2010, 2012, 2013) and Kashirina et al. (2012) are applied to the quasi-one-dimensional case corresponding to the Holstein model of a large-radius polaron.

In recent years increased interest in physics of 1D polarons and 1D bipolarons has been considerably provoked by the development of a lot of new materials, such as metal-oxyde ceramics with layered (La$_2$ (Sr, Br) CuO$_4$ and (Bi, Tl)$_2$ (Sr, Ba)$_2$ CaCuO$_8$) or layered-chain (Y Ba$_2$Cu$_3$O$_7$) structure, demonstrating high-temperature superconductivity (Tohyama 2012; Gunnarsson and Rösch 2008; Moriya and Ueda 2000; Benneman and Ketterson 2008), chain organic (polyacetylene) and inorganic ((SN)$_x$) polymers, quasi-one-dimensional conducting compounds where charge transfer takes place (TTF TCNQ), etc. (Williams et al. 1992; Ishiguro et al. 1998; Toyota et al. 2007; Inzelt 2008; Lebed 2008; Altmore and Chang 2013; Ginzburg 1968; Tulub 1962; Lakhno 2010, 2012; Kashirina et al. 2012; Lakhno 2013; Tohyama 2012; Gunnarsson and Rösch 2008; Moriya and Ueda 2000; Benneman and Ketterson 2008; Schüttler and Holstein 1986; Emin 1986; Kashirina and Lakhno 2015). Much the same as 1D systems can be materials with huge anisotropy where polarons or bipolarons can emerge (Schüttler and Holstein 1986; Emin 1986; Kashirina and Lakhno 2015). Development of DNA-based nanobioelectronics (Lakhno 2008; Offenhüsser and Rinaldi 2009) is also closely related with calculation of polaron and bipolaron properties in one-dimensional molecular chains (Basko and Conwell 2002; Fialko and Lakhno 2000; Conwell and Rakhmanova 2000; Lakhno and Sultanov 2012). Despite great theoretical efforts, many problems of polaron physics have not been solved yet.

One of the central problems of polaron physics is that of spontaneous breaking of symmetry of the “electron + lattice” system. In most papers on polaron physics, following initial Landau hypothesis (Landau 1933) valid for classical lattice, (see books and reviews Pekar 1963; Kuper and Whitfield 1963; Firsov 1975; Devreese and Peeters 1984; Lakhno 1994; Devreese and Alexandrov 2009; Emin 2013; Kashirina and Lakhno 2013) it is thought that at rather a large coupling an electron deforms a lattice so heavily that it becomes self-trapped in the deformed region. In this case the initial symmetry of the Hamiltonian is broken: an electron passes on from the delocalized state having the Hamiltonian symmetry to the localized “self-trapped” state with broken symmetry. This problem is still more actual for bipolarons since a bipolaron state can arise only in the case of large values of the coupling constant.

As showed in Lakhno (2014) for 1D Holstein polaron in a continuum limit for all the values of the coupling constant, the minimum of its energy in quantum lattice is reached in the class of delocalized wave functions. So in Lakhno (2014) it is shown that in the case of a strong-coupling polaron, symmetry is not broken and a self-trapped state is not formed.

In this paper the results of paper Lakhno (2014) are generalized to the case of 1D bipolaron.

In section “Bipolarons with broken translation invariance in the Holstein model in the strong coupling limit” we present known exact results for a polaron and bipolaron with broken translation invariance in the Holstein continuum model in the strong coupling limit when Coulomb interaction between electrons is lacking. In the general case, when the Coulomb interaction takes place, the properties of the bipolaron ground state are illustrated with the use of a variational approach in which the localized wave function of the exact solution without Coulomb interaction is used as a probe one. The results obtained are used to present the criteria of the bipolaron stability.

In section “Translation invariant bipolaron theory” a translation invariant bipolaron theory is constructed. The wave function of such a bipolaron is delocalized. In the strong coupling limit the functional of the bipolaron total energy is derived.

In section “Variational calculation of the bipolaron state” to study the minimum of the total energy a direct variational method is used. It is shown that, as distinct from a bipolaron with broken symmetry, a translation invariant bipolaron exists for all the values of the Coulomb repulsion constant. The regions where a translation invariant bipolaron is stable relative to its decay into two individual polarons are found. It is shown that for all the values of the Coulomb repulsion parameter, the energy of a translation invariant bipolaron is lower than that of a bipolaron with spontaneously broken symmetry.

In section “Spectrum of excited states” we analyze solutions of the equations for the translation invariant bipolaron (below TI-bipolaron) spectrum. It is shown that the spectrum has a gap separating the ground state of a TI-bipolaron from its excited states which form a quasicontinuous spectrum. The concept of an ideal gas of TI-bipolarons is substantiated.

With the use of the spectrum obtained, in section “Statistical thermodynamics of 1D gas of TI-bipolarons” we consider thermodynamic characteristics of an ideal gas of TI-bipolarons. For various values of the parameters, namely phonon frequencies, we calculate the values of critical temperatures of Bose condensation, latent heat of transition into the condensed state, heat capacity and heat capacity jumps at the point of transition.

In section “Comparison with discrete model” we compare the results for continuum and discrete models.

In section “Discussion of results” we discuss the results obtained.

## Bipolarons with broken translation invariance in the Holstein model in the strong coupling limit

According to Lakhno (2006, 2014) and Holstein (1959), Holstein Hamiltonian in a one-dimensional chain in a continuum limit has the form:

1 $$\begin{aligned} H &= -\frac{1}{2m}\Delta _{x_{1}}-\frac{1}{2m}\Delta _{x_{2}}+ \sum _{k}\left[ V_{k}\left( e^{ikx_1}+e^{ikx_2}\right) a_k+h.c.\right] \\&\quad +\,\sum _k\hbar \omega ^0_ka^+_ka_k+U\left( x_1-x_2\right) ,\quad V_k=\frac{g}{\sqrt{N}},\quad \omega ^0_k=\omega _0, \end{aligned}$$

where $a^+_k, a_k$ are operators of the phonon field, *m* is the electron effective mass, $\omega ^0_k$ is the frequency of optical phonons, *g* is the constant of electron-phonon interaction, *N* is the number of atoms in the chain, $U(x_1-x_2)$ is the Coulomb repulsion between electrons depending on the difference of electron coordinates which will be taken to be:

2 $$U\left( x_1-x_2\right) = \varGamma \delta \left( x_1-x_2\right)$$

where $\varGamma$ is a certain constant, $\delta (x)$ is a delta function. In the case of broken translation invariance the bipolaron state is described by localized wave functions $\varPsi = \varPsi (x_1,x_2)$ and in the strong coupling limit the functional of the total energy $\bar{H}=\left\langle \varPsi \left| H\right| \varPsi \right\rangle$ is written as Kashirina and Lakhno (2010):

3 $$\begin{aligned} \bar{H} &= -\frac{1}{2m}\sum _{i=1,2}\left\langle \varPsi \left| \Delta _{x_i}\right| \varPsi \right\rangle - \sum \frac{V^2_k}{\hbar \omega _0}\left\langle \varPsi \left| e^{ikx_1}+e^{ikx_2}\right| \varPsi \right\rangle ^2 \\&\quad +\,\left\langle \varPsi \left| U\left( x_1-x_2\right) \right| \varPsi \right\rangle. \end{aligned}$$

The exact solution of problem (3) is a complicated computational problem (Kashirina and Lakhno 2014; Emin et al. 1992). For the purposes of this section, however, it will suffice to illustrate the properties of the ground state of a bipolaron with broken symmetry with the use of a direct variational method. Towards this end let us choose the probe function $\varPsi = \varPsi (x_1,x_2)$ in the form $\varPsi (x_1,x_2)=\varphi (x_1)\varphi (x_2)$. Notice that this choice of the probe function corresponds to the exact solution of problem (3) for $U=0$, i.e. in the absence of the Coulomb interaction between electrons.

As a result, from (3) we get the functional of the ground state energy:

4 $$\bar{H}=\frac{1}{m}\int \left| \nabla _x\varphi (x)\right| ^2dx-\left( \frac{4g^2a_0}{\hbar \omega _0}-\varGamma \right) \int \left| \varphi (x)\right| ^4dx,$$

where $a_0$ is the lattice constant. Variation of (4) with respect to $\varphi (x)$, the normalization requirement being met, leads to Schroedinger equation:

5 $$\frac{\hbar ^2}{m}\Delta _x\varphi + 2\left( \frac{4g^2a_0}{\hbar \omega _0}-\varGamma \right) \left| \varphi \right| ^2\varphi + W\varphi =0,$$

whose solution has the form:

6 $$\begin{aligned} \varphi (x) &= \pm \left( \sqrt{2r}ch\frac{x-x_0}{r}\right) ^{-1},\quad r=\frac{2\hbar ^2}{m}\frac{1}{\left( {(4g^2a_0)}/{(\hbar \omega _0)}-\varGamma \right) }, \\ W &= -\frac{1}{2}\left( \frac{4g^2a_0}{\hbar \omega _0}-\varGamma \right) ^2\frac{m}{2\hbar ^2},\quad E_{bp}=-\frac{1}{6}\left( \frac{4g^2a_0}{\hbar \omega _0}-\varGamma \right) ^2 \frac{m}{2\hbar ^2}, \end{aligned}$$

where $x_0$ is an arbitrary constant, $E_{bp}=min\;\bar{H}$ is the energy of the bipolaron ground state. Notice, that the polaron state energy $E_p$ in the case under consideration is Lakhno (2014):

7 $$E_P=-\frac{1}{6}\left( \frac{g^2a_0}{\hbar \omega _0}\right) ^2\frac{m}{\hbar ^2}.$$

Let us introduce the notation:

8 $$\gamma =\varGamma \hbar \omega _0/a_0g^2.$$

From (6) it follows that for:

9 $$\gamma >4$$

the existence of the bipolaron state is impossible. In the case of:

10 $$2<\gamma <4$$

the metastable bipolaron state will decay into individual polaron states. As:

11 $$\gamma <2$$

the bipolaron state will be stable. Notice that the choice of more complex probe functions Kashirina and Lakhno (2014) has no effect on the qualitative picture presented, changing only the numerical coefficients in relations (9)–(11).

In view of an arbitrary position of the bipolaron center of mass $x_0$, the bipolaron state discussed has an infinite degeneracy and can move along the chain. Any arbitrarily small violation of the chain leads to elimination of the degeneration and localization of the bipolaron state on defects with attracting potential. A qualitatively different situation arises in the case of a translation invariant bipolaron considered below.

## Translation invariant bipolaron theory

To construct a translation invariant bipolaron theory in the Holstein model, in Hamiltonian (1) we pass on to coordinates of the center of mass. In this system Hamiltonian (1) takes the form:

12 $$\begin{aligned} H&=-\frac{\hbar ^2}{2M}\Delta _{R}-\frac{\hbar ^2}{2\mu }\Delta _{r}+ \sum _k 2V_k cos\frac{kr}{2}\left( e^{ikR}a_k+h.c.\right) \\&\quad +\,\sum _k\hbar \omega ^0_k a^+_k a_k+U(r), \\ R&= \left( x_1+x_2\right) /2,\quad r=x_1-x_2,\quad M=2m,\quad \mu =m/2. \end{aligned}$$

In what follows we will use units, putting $\hbar =1, \omega _0=1, M=1$ (accordingly $\mu =1/4$).

The coordinate of the center of mass *R* in Hamiltonian (2) can be eliminated via Heisenberg canonical transformation Heisenberg (1930):

13 $$\hat{S}_1=exp\left\{ -i\sum _kka^+_ka_kR\right\}.$$

As a result, the transformed Hamiltonian: $\tilde{H}=\hat{S}^{-1}_1H\hat{S}_1$ is written as:

14 $$\begin{aligned} \tilde{H}&= -2\Delta _r+\sum _k2V_k cos \frac{kr}{2}\left( a^+_k+a_k\right) + \sum _ka^+_ka_k+U(r) \\&\quad +\,\frac{1}{2}\left( \sum _ka^+_ka_k\right) ^2. \end{aligned}$$

From (14) it follows that the exact solution of the bipolaron problem is determined by the wave function $\varPsi (r)$ which depends only on the relative coordinates *r* and, therefore, is automatically translation invariant. It corresponds to the state delocalized over the coordinates of the center of mass of two electrons.

Averaging Hamiltonian (14) over $\varPsi (r)$, we will write the averaged Hamiltonian as: $\bar{\tilde{H}}$ (15)

15 $$\begin{aligned} \bar{\tilde{H}}&= \bar{T}+\sum _k \bar{V}_k\left( a^+_k+a_k\right) + \sum _ka^+_ka_k+\frac{1}{2}\left( \sum _ka^+_ka_k\right) ^2+\bar{U}, \\ \bar{V}_k&= 2V_k\left\langle \varPsi \left| cos \frac{kr}{2}\right| \varPsi \right\rangle , \quad \bar{U}=\left\langle \varPsi \left| U(r)\right| \varPsi \right\rangle ,\quad \bar{T}=-2\left\langle \varPsi \left| \Delta _r\right| \varPsi \right\rangle .\end{aligned}$$

Subjecting Hamiltonian (15) to Lee–Low–Pines transformation (Lee et al. 1953):

16 $$\hat{S}_2=exp\left\{ \sum _k f_k\left( a_k-a^+_k\right) \right\},$$

we get:

17 $$\tilde{\tilde{H}}=\hat{S}^{-1}_2\bar{\tilde{H}}\hat{S}_2\quad \tilde{\tilde{H}}=H_0+H_1$$

where:

18 $$H_0 = \bar{T}+2\sum _k\bar{V}_kf_k+\sum _kf^2_k+\frac{1}{2}\left( \sum _kkf_k\right) ^2+\bar{U}+{\mathcal{{H}}}_0$$

19 $${\mathcal{{H}}}_0 = \sum _k\omega _ka^+_ka_k+ \frac{1}{2}\sum _{k,k'}kk'f_kf_{k'}\left( a_ka_{k'}+a^+_ka^+_{k'}+a^+_ka_{k'}+a^+_{k'}a_k\right) ,$$

20 $$\begin{aligned} H_1&= \sum _k\left( V_k+f_k\omega _k\right) \left( a_k+a^+_k\right) + \sum _{k,k'}kk'f_{k'}\left( a^+_ka_ka_{k'}+a^+_ka^+_{k'}a_k\right) \\&\quad +\,\frac{1}{2}\sum _{k,k'}kk'a^+_ka^+_{k'}a_ka_{k'}, \end{aligned}$$

21 $$\omega _k = \omega _0+\frac{k^2}{2}+k\sum _{k'}k'f^2_{k'}.$$

According to Tulub (1962), contribution of $H_1$ into the energy vanishes if the eigen function of Hamiltonian ${\mathcal{{H}}}_0$ transforming the quadratic form ${\mathcal{{H}}}_0$ to the diagonal one, is chosen properly. Diagonalisation of ${\mathcal{{H}}}_0$ leads to the total energy of the addition $\Delta E$:

22 $$\Delta E=\frac{1}{2}\sum _{k}\left( \nu _k-\omega _k\right) =-\frac{1}{8\pi i}\int _{c}\frac{ds}{\sqrt{s}}ln D(s),$$

where $\nu _k$ are phonon frequencies renormalized by the interaction with the electron. The contour of integration *c* involved in (22) is the same as in Tulub (1962) and Lakhno (2014). In the one-dimensional case under consideration:

23 $$D(s)=1-\frac{1}{\pi }\int ^{\infty }_{-\infty }\frac{k^2f_k\omega _k}{s-\omega ^2_k}dk.$$

Repeating calculations carried out in Tulub (1962) and Lakhno (2014) in the strong coupling limit, we express $\Delta E$ as:

24 $$\begin{aligned} \Delta E&= \frac{1}{4\pi }\int ^{\infty }_{-\infty }\frac{k^2f^2_kdk}{2(1+Q)} \\&\quad+\,\frac{1}{4\pi ^2}\iint ^{\infty }_{-\infty }\frac{k^2f^2_kp^2f^2_p\omega _p(\omega _k\omega _p+\omega _k(\omega _k+\omega _p))+1}{(\omega _k+\omega _p)^2(\omega ^2_p-1)\left| D_+(\omega ^2_p)\right| ^2}dpdk,\\ D_+(\omega ^2_p) &= 1+\frac{1}{\pi }\int ^{\infty }_{-\infty }\frac{f^2_kk^2\omega _kdk}{\omega ^2_k-\omega ^2_p-i\epsilon }, \\ Q&= \frac{1}{\pi }\int ^{\infty }_{-\infty }\frac{k^2f^2_k\omega _kdk}{\omega ^2_k-1}. \end{aligned}$$

Finally, with the use of (18) and (19) the bipolaron total energy $E_{bp}$ is written as:

25 $$E_{bp}=\Delta E+2\sum _k\bar{V}_kf_k+\sum _kf^2_k+\bar{T}+\bar{U} .$$

## Variational calculation of the bipolaron state

We could have derived an exact equation for determining the bipolaron energy by varying (25) with respect to $\varPsi$ and $f_k$. The quantities $\varPsi$ and $f_k$ obtained as solutions of this equation, being substituted into (25) determine the bipolaron total energy $E_{bp}$. Since finding a solution of the equation obtained by variation of $E_{bp}$ is rather a complicated procedure, we will use the variational approach. To this end let us choose the probe functions $\varPsi$ and $f_k$ in the form:

26 $$\varPsi (r) = \left( \frac{2}{\pi }\right) ^{1/4}\frac{1}{\sqrt{l}}e^{-r^2/l^2},$$

27 $$f_k = -Nge^{-k^2/2a^2},$$

where *N*, *l*, *a* are variational parameters. As a result, after minimization of (25) on *N*, the bipolaron energy will be:

28 $$E_{bp} = \frac{ma^{2}_{0}}{\hbar ^2}\frac{g^4}{\hbar ^2\omega ^{2}_{0}}\;{min}_{(x,y)}E\left( x,y;\gamma \right) ,$$

29 $$E(x,y;\gamma )\approx {} 2\left( 0.390625x^2+\frac{2}{y^2}-\frac{4x}{\sqrt{\pi }(1+x^2y^2/16)}+\sqrt{\frac{2}{\pi }}\frac{\gamma }{y}\right)$$

**Fig. 1:**
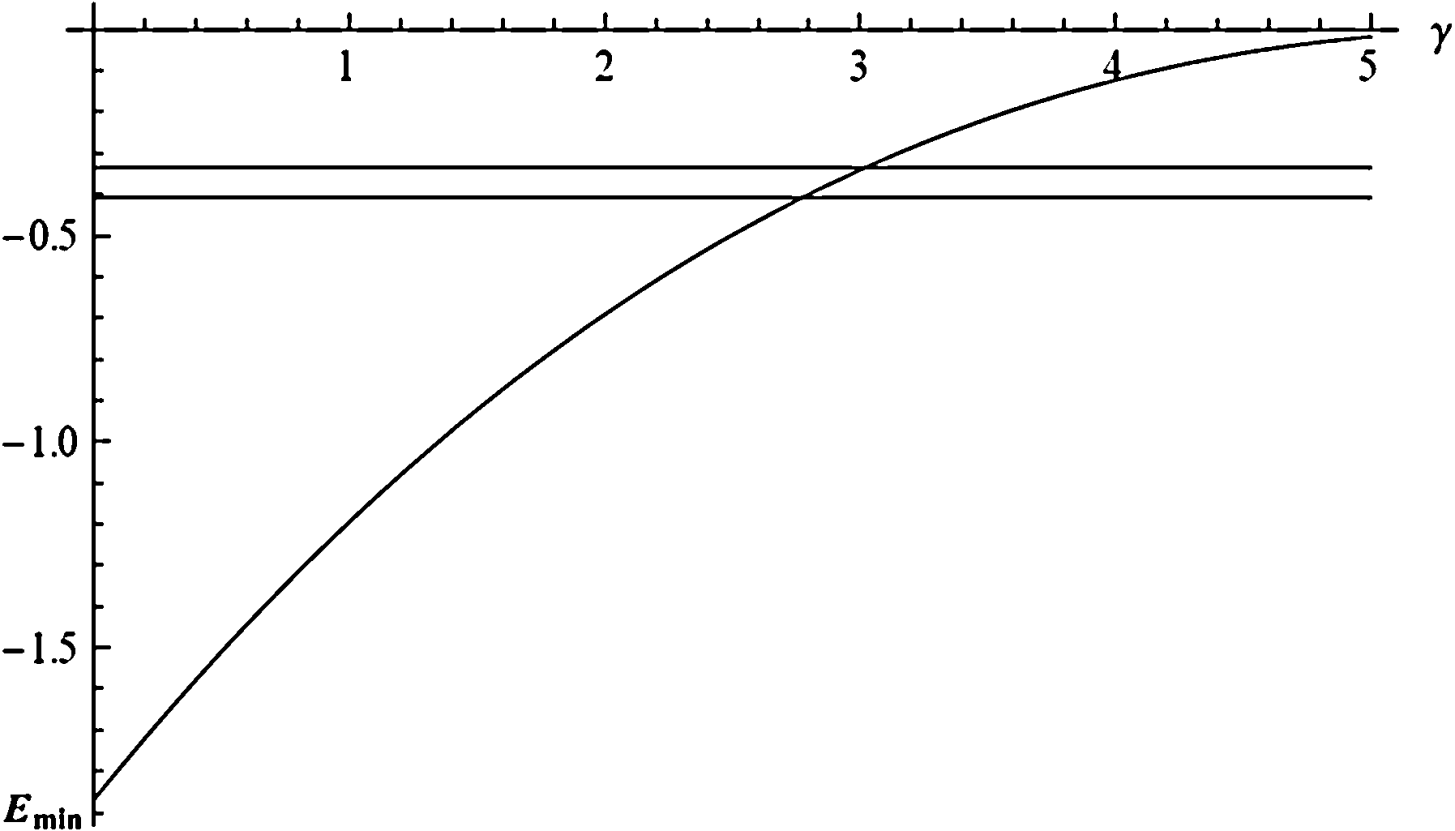
The dependence of $E_{min}$ (29) on $\gamma$

The expression for the bipolaron energy is given in dimension units. The results of minimization of function $E(x,y;\gamma )$ with respect to dimensionless parameters *x*, *y* are presented in Fig. 1 for various values of the parameter $\gamma$. Figure 1 suggests that as distinct from a bipolaron with broken symmetry [inequality (9)], a translation invariant bipolaron exists for all the values of the parameter $\gamma$. In the region:

30 $$\gamma > 3.02$$

a translation invariant bipolaron is unstable relative to its decay into both individual polarons with spontaneously broken symmetry, i.e. Holstein polarons with the energy $2E_p=-(1/3)ma^2_0g^4/\hbar ^4\omega ^2_0$ (upper horizontal line in Fig. 1 in energy units $ma^2_0g^4/\hbar ^4\omega _0$) and translation invariant polarons with the energy $2E_p=-0.4074ma^2_0g^4/\hbar ^4\omega ^2_0\,$ (Lakhno 2014) (lower horizontal line in Fig. 1). For:

31 $$2.775< \gamma <3.02$$

a translation invariant bipolaron becomes stable relative to its decay into individual Holstein polarons, but remains unstable relative to decomposition into individual translation invariant polarons. For:

32 $$\gamma < \gamma _c=2.775$$

a translation invariant bipolaron becomes stable relative to its decay into two individual polarons. Notice that for $\gamma =0$, the energy of a translation invariant bipolaron is equal to: $E_{bp}=-1.87104\,ma^2_0g^4/\hbar ^4\omega ^2_0$, i.e. lies much lower than the exact value of the energy of a bipolaron with broken symmetry, which, according to (6) is equal to $E_{bp}=-(4/3)ma^2_0g^4/\hbar ^4\omega ^2_0$. The energy of a translation invariant bipolaron also lies below the variational estimate of the energy of a bipolaron with spontaneously broken symmetry (6) for all the values of $\gamma$ (Kashirina and Lakhno 2014).

The dimensionless parameters *x*, *y* involved in (29) are related to the variational parameters *a* and *l* (26) and (27) as: $a=(2ma^2_0g^2/\hbar ^3\omega _0)x, l=(\hbar ^3\omega _0/2ma^2_0g^2)y$. The parameter *l* determine the characteristic size of the electron pair, i.e. the correlation length $L(\gamma )$, whose dependence on $\gamma$ is given by the expression:

33 $$L(\gamma )=\frac{\hbar ^2}{2ma^{2}_{0}}\frac{\hbar \omega _0}{g^2}y_{min} (\gamma ).$$

**Fig. 2:**
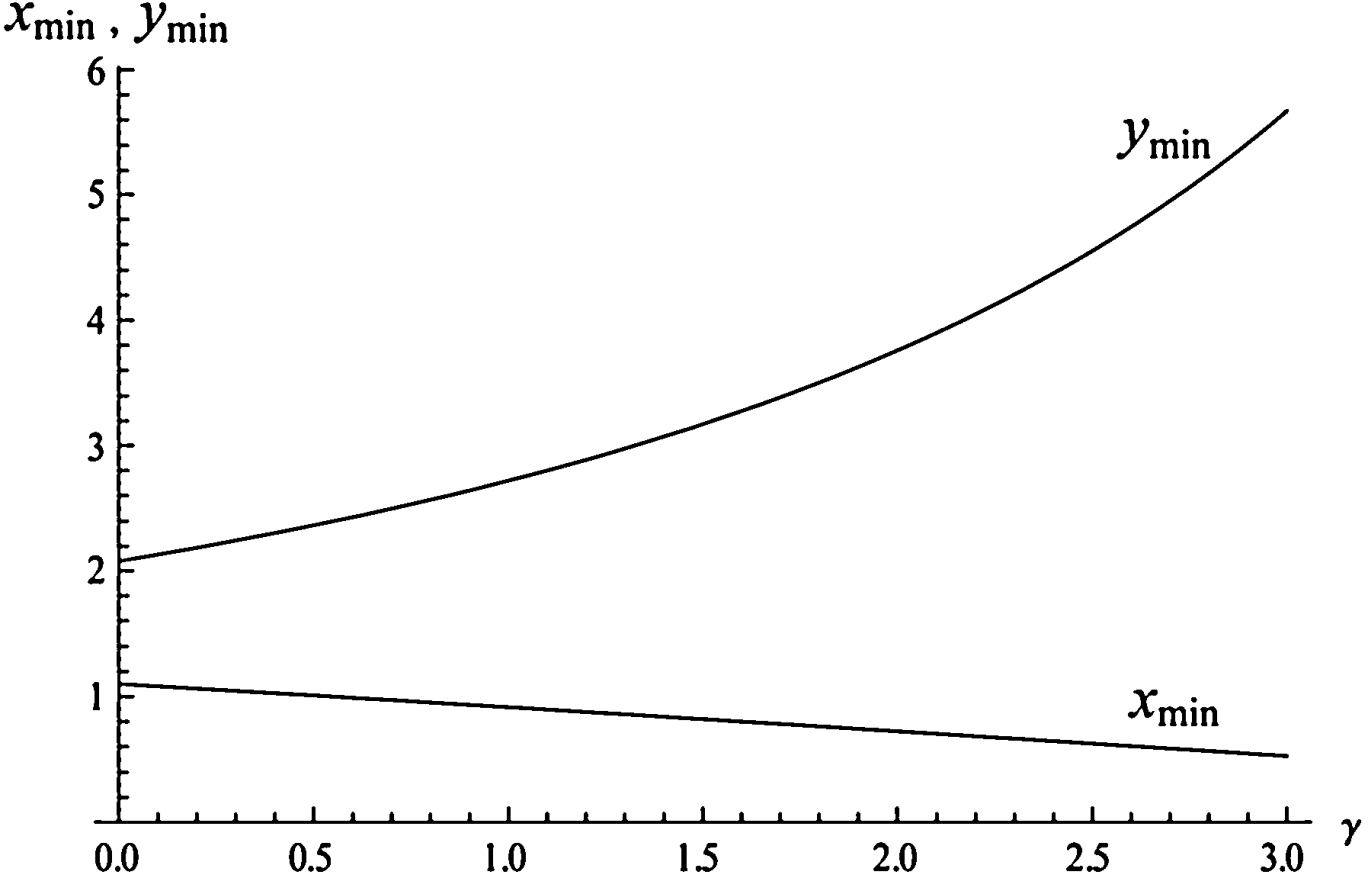
The dependence of $x_{min}, y_{min}$ on $\gamma$

The dependencies of $y_{min}$ and $x_{min}$ on $\gamma$ are presented in Fig. 2.

Figure 2 suggests that the correlation length $L(\gamma )$ in the region of a bipolaron stability $0<\gamma <\gamma _c$ does not change greatly and for its critical value $\gamma _c=2.775$ the quantity $L(\gamma )$ approximately three times exceeds the value of *L*(0), i.e. the correlation length in the absence of the Coulomb repulsion. This qualitatively differs from the case of a bipolaron with broken symmetry for which the corresponding value, according to (6), for $\gamma =\gamma _c$ turns to infinity.

## Spectrum of excited states

According to the results obtained in Tulub (1962) and Lakhno (2015b), the spectrum of excited states of Hamiltonian (18) and (19) is determined by the expression:

34 $$\tilde{\tilde{H}} = E_{bp}+\sum _k\nu _k\alpha ^+_k\alpha _k$$

where $\alpha ^+_k, \alpha _k$ are operators in which quadric form $H_0$ (19) is diagonal. Operators $\alpha ^+_k, \alpha _k$ can be considered as operators of birth and annihilation of TI-bipolarons in excited states obeying Bose commutation relations:

35 $$\left[ \alpha _n,\alpha ^+_{n'}\right] = \alpha _n\alpha ^+_{n'} - \alpha ^+_n\alpha _n =\delta _{n,n'}$$

Renormalized frequencies involved in (34), according to Tulub (1962) and Lakhno (2015b), are determined by the equation for *s*:

36 $$1=2\sum _k\frac{k^2f_k\omega _k}{s-\omega ^2_k}$$

solutions of which give the spectrum of $s=\{\nu ^2_k\}$ solutions.

It is convenient to present Hamiltonian (34) in the form:

37 $$\tilde{\tilde{H}}= \sum _{n=0,1,2}E_n\alpha ^+_n\alpha _n$$

38 $$E_n ={\left\{ \begin{array}{ll} E_{bp}, &{} \quad n=0; \\ \nu _n=E_{bp}+\omega _0+\frac{k^2_n}{2}, &{} \quad n \ne 0. \end{array}\right. }$$

where $k_n$ for a discrete chain of atoms is equal to:

$$k_n=\pm \frac{2\pi (n-1)}{N_a},\quad n=1,2,\ldots ,N_a/2+1$$

$N_a$ is the number of atoms in the chain.

Let us prove the validity of (38). The energy spectrum of TI-bipolarons, according to (36), reads:

39 $$F(s) = 1$$

40 $$F(s) = {} 2\sum _n\frac{k^2_nf^2_{k_n}\omega _{k_n}}{s-\omega ^2_{k_n}}$$

**Fig. 3:**
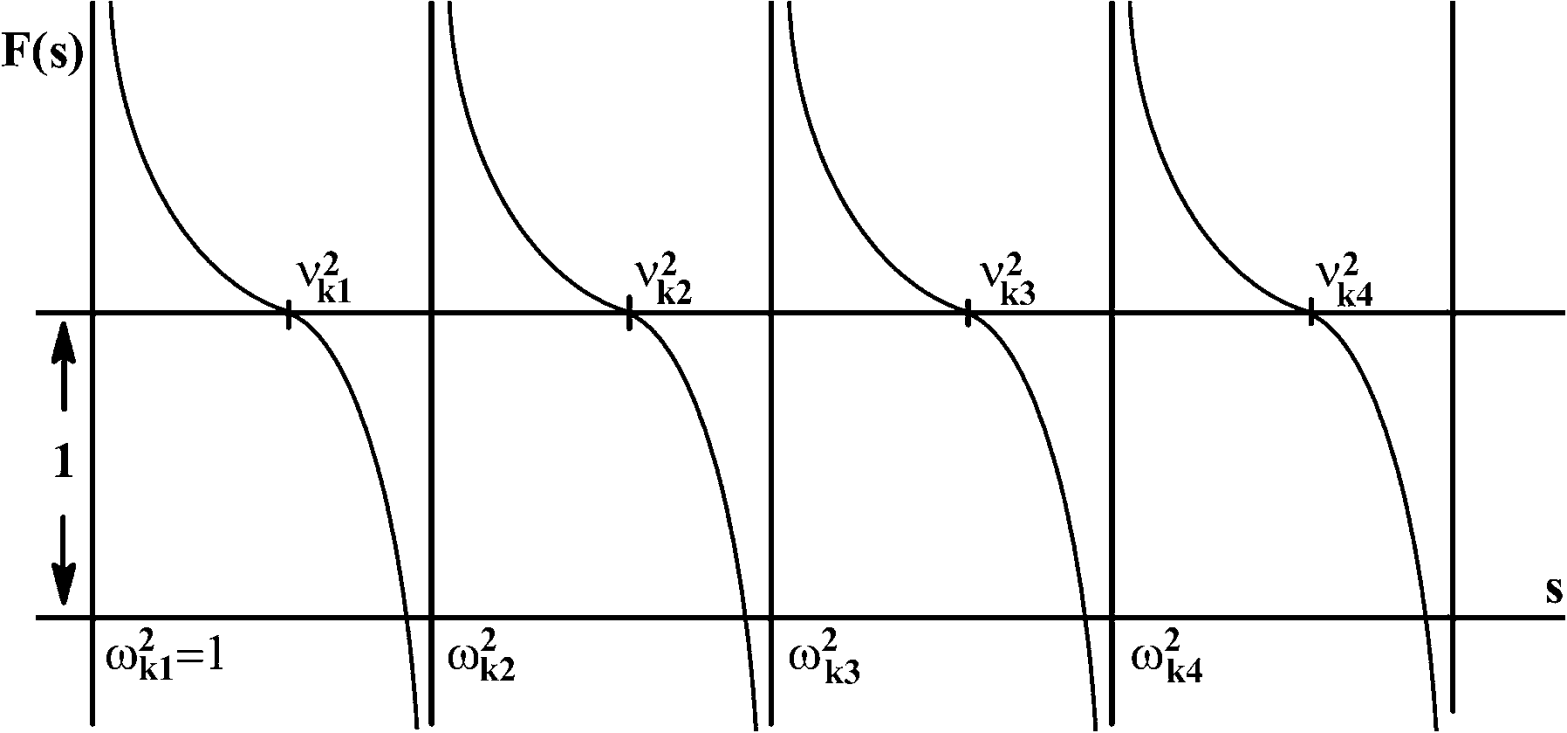
Graphical solution of Eqs. (39) and (40)

It is convenient to solve Eq. (39) graphically (Fig. 3).

Figure 3 suggests that frequencies $\nu _{k_n}$ occur between the frequencies $\omega _{k_n}$ and $\omega _{k_{n+1}}$. Hence, the spectrum of $\nu _{k_n}$ as well as the spectrum of $\omega _{k_n}$ is quasi continuous in the continuum limit: $\nu _{k_n}-\omega _{k_n}=0(N^{-1}_a)$, which proves the validity of (37) and (38).

Therefore the spectrum of a TI-bipolaron has a gap between the ground state of $E_{bp}$ and the quasi continuum spectrum, which is equal to $\omega _0$.

Below we will consider the case of low concentration of TI-bipolarons in the chain. In this case they can be adequately considered as Bose-gas, whose properties are determined by Hamiltonian (37).

## Statistical thermodynamics of 1D gas of TI-bipolarons

Let us consider the rare (the pair correlation length is much smaller then the average distance between pairs) one-dimensional ideal Bose-gas of TI-bipolarons which is a system of *N* particles, occurring in a one-dimensional chain of length *L*. Let us write $N_0$ for the number of particles in the lower one-particle state, and *N* for the number of particles in higher states. Then:

41 $$N= {} \sum _{n=0,1,2,\ldots }\bar{m}_n=\sum _n\frac{1}{e^{(E_n-\mu )/T}-1}$$

42 $$N= {} N_0+N',\quad N_0=\frac{1}{e^{(E_0-\mu )/T}-1},\quad N'=\sum _{n \ne 0}\frac{1}{e^{(E_n-\mu )/T}-1}$$

**Fig. 4:**
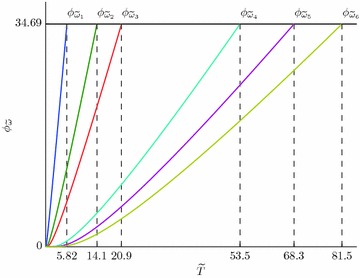
Solutions of Eq. (43) $C_{1D}=34.69$ and $\tilde{\omega }_i=\left\{ 0.2;1;2;10;15;20\right\}$, which correspond to $\tilde{T}_{c_i}=\tilde{T}_{c_1}=5.823; \tilde{T}_{c_2}=14.1; \tilde{T}_{c_3}=20.87; \tilde{T}_{c_4}=53.47; \tilde{T}_{c_5}=68.33; \tilde{T}_{c_6}=81.5$

In expression for $N'$ (42) we will replace summation by integration over quasi continuous spectrum (37) and (38) and take $\mu =E_{bp}$. As a result we will get from (41) and (42) an expression for the temperature of Bose condensation $T_c$:

43 $$C_{1D}=\varPhi _{\tilde{\omega }}(T_c)$$

$$\begin{aligned} \varPhi _{\tilde{\omega }}&= {} \tilde{T}^{1/2}_{c}F_{1/2}\left( \frac{\tilde{\omega }}{\tilde{T}_c}\right) ,\quad F_{1/2}(\alpha )=\int _0^{\infty }\frac{dx}{\sqrt{x}\left( e^{x+\alpha }-1\right) }, \\ C_{1D}&= {} 2\sqrt{2}\pi \frac{n\hbar }{M^{1/2}\omega ^{*1/2}},\quad \omega ^*=\frac{\omega _0}{\omega }, \tilde{T}_c=\frac{T}{\omega ^*}, \end{aligned}$$

where $n=N/L$. Figure 4 shows a graphical solution of Eq. (43) for the parameter values: $M_e=2m=2m_0$, where $m_0$ is the mass of a free electron in vacuum, $\omega ^*=5$ meV, (${\approx}58$ K), $n=10^7$ cm$^{-1}$ and the values: $\tilde{\omega }_1=0.2; \tilde{\omega }_2=1; \tilde{\omega }_3=2; \tilde{\omega }_4=10; \tilde{\omega }_5=15; \tilde{\omega }_6=20$ ($C_{1D}=34.69$).

Figure 4 suggests that the critical temperature grows as the phonon frequency increases and is equal to zero for $\omega =0$. The equality $T_c=0$ for $\omega =0$ corresponds to the known result, that Bose-condensation is impossible in ideal gas in a one-dimensional case.

Figure 4 also suggests that it is just the increase in the concentration of TI-bipolarons which will lead to an increase in the critical temperature, while the increase in the electron mass *m* to its decrease.

It follows from (41) and (42) that:

44 $$\frac{N'(\tilde{\omega })}{N} = {} \frac{\tilde{T}^{1/2}}{C_{1D}}F_{1/2}\left( \frac{\tilde{\omega }}{\tilde{T}}\right)$$

45 $$\frac{N_0(\tilde{\omega })}{N}= {} 1-\frac{N'(\tilde{\omega })}{N}$$

**Fig. 5:**
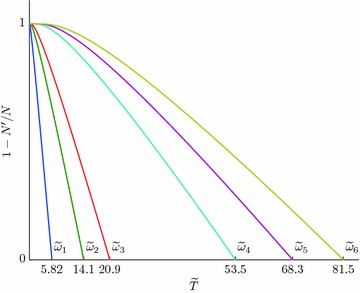
Temperature dependencies of the relative number of supracondensate particles $N'/N$ and condensate particles $N_0/N$ for the values of $\tilde{\omega }_i$, given in Fig. 4

Figure 5 illustrates temperature dependencies of the supracondensate particles $N'$ and the particles in the condensate $N_0$ for the above-cited values of $\tilde{\omega }_c$ parameters.

From Fig. 5 it follows that, as we might expect, the number of particles in the condensate grows as the gap $\omega _i$ increases.

The energy of TI-bipolaron gas *E* reads:

46 $$E=\sum _{n=0,1,2,\ldots }\bar{m}_nE_n=E_{bp}N_0+\sum _{n\ne 0}\bar{m}_nE_n$$

With the use of (37) and (38) for the specific energy (i.e. energy per one TI-bipolaron) $\tilde{E}(\tilde{T})=E/N\omega ^*, \tilde{E}_{bp}=E_{bp}/\omega ^*$ (46) transforms into:

47 $$\tilde{E}= {} \tilde{E}_{bp}+\Delta \tilde{E}$$

48 $$\begin{aligned} \Delta \tilde{E}&= {} \frac{\tilde{T}^{3/2}}{C_{1D}}F_{1/2}\left( \frac{\tilde{\omega }-\tilde{\mu }}{\tilde{T}}\right) \left[ \frac{\tilde{\omega }}{\tilde{T}}+\frac{F_{3/2}\left( \frac{\tilde{\omega }-\tilde{\mu }}{\tilde{T}}\right) }{F_{1/2}\left( \frac{\tilde{\omega }-\tilde{\mu }}{\tilde{T}}\right) }\right] , \end{aligned}$$

49 $$F_{3/2}(\alpha ) = {} \int ^{\infty }_0\frac{\sqrt{x}dx}{e^{x+\alpha }-1}$$

where $\mu$ is determined by the equation:

50 $$C_{1D}=\tilde{T}^{1/2}_c F_{1/2}\left( \frac{\tilde{\omega }-\tilde{\mu }(\tilde{T})}{\tilde{T}}\right)$$

$$\tilde{\mu }= {\left\{ \begin{array}{ll} 0, &{} \quad\tilde{T}<\tilde{T}_c; \\ \tilde{\mu }(\tilde{T}), &{} \quad\tilde{T}>\tilde{T}_c. \end{array}\right. }$$

Relation between $\tilde{\mu }$ and the chemical potential of the system $\mu$ is given by the expression $\tilde{\mu }=(\mu -E_{bp})/\omega ^*$. Formulae (49) and (50) also yield expressions for $\varOmega$—potential: $\varOmega =-2E$ and entropy $S=-\partial \varOmega /\partial T$ ($F=-2E, S=-\partial F/\partial T$).

**Fig. 6:**
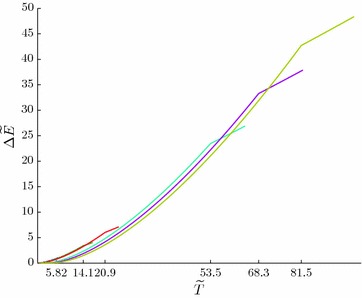
The temperature dependencies $\Delta \tilde{E} =\tilde{E}(\tilde{T})-\tilde{E}_{bp}$ for various values of $\tilde{\omega }_i$ (see Table 1)

Figure 6 demonstrates temperature dependencies of $\Delta \tilde{E} =\tilde{E}-\tilde{E}_{bp}$ for the above-cited values of $\tilde{\omega }_i$. Salient points of $\Delta \tilde{E}_i(\tilde{T})$ curves correspond to the values of critical temperatures $T_{c_i}$.

These dependencies enable us to find the heat capacity of TI-bipolaron gas: $C_V(\tilde{T})=d\tilde{E}/d\tilde{T}$.

Figrue 7 shows temperature dependencies of the heat capacity $C_V(\tilde{T})$ for the above-cited values of $\tilde{\omega }_i$. Table 1 lists the heat capacity jumps for the values of parameters $\tilde{\omega }_i$:

51 $$\Delta \frac{\partial C_V(\tilde{T})}{\partial \tilde{T}}={\frac{\partial C_V(\tilde{T})}{\partial \tilde{T}}}\Biggl |_{\tilde{T}=\tilde{T}_c+0}\Biggr . -{\frac{\partial C_V(\tilde{T})}{\partial \tilde{T}}}\Biggl |_{\tilde{T}=\tilde{T}_c-0}\Biggr.$$

at the transition points.

**Fig. 7:**
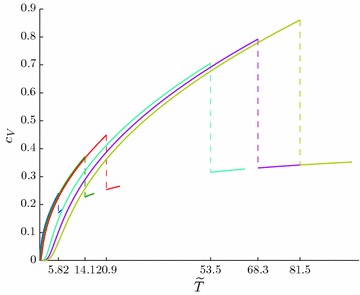
The temperature dependencies of the heat capacity for various values of $\tilde{\omega }_i$ (see Table 1)

The dependencies obtained enable us to find the latent heat of the transition $q=TS$, where *S* is the entropy of supracondensate particles. At the transition point this value is $q=2T_cC_V(T_c-0)$,$C_V=d\tilde{E}/d\tilde{T}$ and $\tilde{E}$ is determined by formulae (47) and (48). The values of the heat of transition $q_i$ for the above-cited values of $\tilde{\omega }_i$ are given in Table 1.

**Table 1:** Dependence of critical temperatures $\tilde{T}_{c_i}$, heat capacities $C_V(\tilde{T}_{c_i}\pm 0)$, and heat capacity jumps $\Delta$ on the values of $\tilde{\omega }_i$

*i*	1	2	3	4	5	6
$\tilde{\omega }_i$	0.2	1	2	10	15	20
$\left. \tilde{T}_{c_i}\right.$	5.82	14.11	20.87	53.47	68.33	81.5
$C_V(\tilde{T}_{c_i}-0)$	0.24	0.37	0.45	0.7	0.79	0.86
$C_V(\tilde{T}_{c_i}+0)$	0.17	0.23	0.25	0.32	0.33	0.34
$\frac{\partial C_V}{\partial \tilde{T}}(\tilde{T}_{c_i}-0)$	$5.23\times 10^{-3}$	$-0.93\times 10^{-3}$	$-2.64\times 10^{-3}$	$-4.94\times 10^{-3}$	$-5.2\times 10^{-3}$	$5.34\times 10^{-3}$
$\frac{\partial C_V}{\partial \tilde{T}}(\tilde{T}_{c_i}+0)$	$10.22\times 10^{-3}$	$4.72\times 10^{-3}$	$3.24\times 10^{-3}$	$1.19\times 10^{-3}$	$0.89\times 10^{-3}$	$0.73\times 10^{-3}$
$\Delta$	$5.0\times 10^{-3}$	$5.65\times 10^{-3}$	$5.88\times 10^{-3}$	$6.12\times 10^{-3}$	$6.1\times 10^{-3}$	$6.06\times 10^{-3}$

## Comparison with discrete model

Earlier we considered the problem of symmetry breakdown for one electron interacting with oscillations of a one-dimensional quantum chain (Lakhno 2014). According to Lakhno (2014), a rigorous quantum-mechanical treatment leads to delocalized translation-invariant electron states, or to a lack of soliton-type solutions, breaking the initial symmetry of the Hamiltonian.

In this paper we have shown that when the chain contains two electrons which interact with its oscillations and suffer Coulomb repulsion determined by the interaction, a stable state can be formed which does not violate translation invariance and has a lower energy than the localized solution which breaks TI symmetry does.

Presently most papers describing electron states in discrete molecular chains are based on Holstein–Hubbard Hamiltonian (Holstein 1959; Hubbard 1963; Proville and Aubry 1998; Korepin and Eßler 1994):

52 $$\begin{aligned} H&= {} \eta \sum _{i,j,\sigma ,\sigma '}c^+_{i\sigma }c_{j\sigma '}+\sum _i\hbar \omega \left( a^+_ja_j+1/2\right) + \sum _jg\hat{n}_j\left( a^+_j+a_j\right) \\&\quad +\,\sum _{j,\sigma ,\sigma '}U\hat{n}_{j\sigma }\hat{n}_{j\sigma '}, \end{aligned}$$

where $\hat{n}_{j\sigma }=c^+_{j\sigma }c_{j\sigma '}, \hat{n}_j=\sum _{\sigma }\hat{n}_{j\sigma }, c^+_{j\sigma }, c_{j\sigma }$—are operators of the birth and annihilation of an electron with spin $\sigma$ at the jth site; $\eta$—is the matrix element of the transition between nearest sites (*i*, *j*).

Numerical investigations of Hamiltonian (52) are based on the use of ansatz for the wave functions of the ground state:

53 $$\left| \varPsi \right\rangle =\sum _{i,j,\sigma ,\sigma '}\varPsi _{ij}c^+_{i\sigma }c^+_{j\sigma '}\left| 0\right\rangle,$$

where $\left| 0\right\rangle$—is a vacuum wave function which is a product of electron and lattice vacuum functions.

Hamiltonian (1) considered in this work is a continuum analog of Hamiltonian (52) if in (52) we put: $m=\hbar ^2/2\eta a^2_0, \varGamma =Ua_0$. As is shown in Lakhno (2015b), presentation of the wave function as a product of the electron wave function by the lattice one (Pekar ansatz) does not give an exact solution of Hamiltonian (1). A similar conclusion is valid for Hamiltonian (52). In this context it would be interesting to discuss the limits of applicability of ansatz (53) in a discrete case using a particular example.

By way of example of a discrete model let us take the results of calculation of bipolaron states in a Poly G/Poly C nucleotide chain given in paper Lakhno and Sultanov (2011). Table 2 lists the values of the coupling energy $\Delta =|E_{bp}-2E_p|$ in the case of $U=0, \eta =0.084$ eV for a discrete model (52) with using ansatz (53): $\Delta = \Delta ^d$; for a continuum Holstein bipolaron with broken symmetry: $\Delta = \Delta ^H$ (6)–(7); for a continuum TI-bipolaron: $\Delta = \Delta ^{TI}$ (28). These results suggest that $\Delta ^H$ virtually coincides with $\Delta ^d$ and becomes less than $\Delta ^d$ as $\kappa \le 0.1$ gets larger. On the contrary, the values of $\Delta ^{TI}$ for $\kappa \le 0.3$ exceed the values of $\Delta ^d$ and become less than $\Delta ^d$ as gets larger. In the general case we can say that discreteness violates continual translation invariance of the chain only when some threshold value of the coupling constant is exceeded. In particular, for the discrete model of a Poly G/Poly C chain Lakhno and Sultanov (2011) with parameters $U \approx 1$ eV, $\kappa =4g^2/\hbar \omega =0.5267$ which correspond, according to (8), to $\gamma \approx 7.6$, the TI-bipolaron states considered in the paper are probably unstable, since they do not fall on the stability interval $\gamma < \gamma _c$ (32). In this case the states should be calculated based on a discrete model. For DNA, such a calculation, as applied to the possibility of superconductivity in DNA was carried out in papers (Lakhno and Sultanov 2012), Lakhno and Sultanov (2011). It should also be noted that apart from the condition $\gamma < \gamma _c$, for continuum TI-bipolarons to exist, the condition of continuity should also be met. According to Lakhno (2015b) it implies that the characteristic phonon vectors making the main contribution into the energy of TI-bipolarons should satisfy the inequality $ka_0<1$. From (27) it follows that the main contribution into the energy is given by the values of $k \le a$. For $U=0$, this yields $k \le g^2/\hbar \omega \nu a_0$. Accordingly, the condition of continuity takes on the form:

54 $$g^2/\hbar \omega \nu \le 1$$

Obviously, for $U=0$, this condition is equivalent to the requirement $r/a_0\ge 1$, where *r* is determined by (6). From (6) it also follows that for $U \ne 0$ Holstein polaron becomes lengthier, since its characteristic size becomes equal to $r = r_0(1-\gamma /4)^{-1}$, where $r_0$—is the characteristic size for $U=0$. For a TI-bipolaron, the same conclusion follows from expression (33) for the correlation length and Fig. 2. Physically this is explained by the fact that Coulomb repulsion leads to an increase of the characteristic distance between the electrons in the bipolaron state. Earlier this result was also obtained in Emin et al. (1992). Hence, though TI-bipolarons are delocalized, the requirements of continuity for TI-bipolaron and Holstein bipolaron turn out to be similar.

**Table 2:** Coupling energies $\Delta$ for $U=0$ for a discrete model $\Delta ^d$, for continuum Holstein model $\Delta ^H$, and translation invariant bipolaron $\Delta ^{TI}$

$\Delta$	$\kappa$				
	0.1	0.1975	0.296	0.359	0.5267
$\Delta ^d$	0.0037	0.015	0.05	0.112	0.203
$\Delta ^H$	0.0037	0.0145	0.033		
$\Delta ^{TI}$	0.0056	0.022	0.0495		

Table 2 lists the values of $\Delta$ for which the continuum model is more preferable than the ‘exact’ discrete one.

The results obtained suggest that for parameter values when the continuum model is valid and conditions of strong coupling are met, TI-bipolarons are energetically more advantageous. Therewith the question of the character of a transition from the continuum description to the discrete one remains open. One would expect that such a transition will occur with a sharp increase in the bipolaron effective mass as a result of which the molecular chain will change from highly conducting state to low conducting one.

## Discussion of results

The estimate of the value of the coupling constant $g_c=g/\hbar \omega _{0}$ sufficient for the formation of translation-invariant bipolaron states in the region where the criterion of their existence is met $0< \gamma < \gamma _c$ can be obtained by comparing the total energy of a strong coupling bipolaron with twice energy of individual weak coupling polarons. Weak coupling polarons, by their treatment per se (perturbation theory) are translation invariant with the energy (Lakhno 2006):

$$E_p = -g^2\sqrt{ma^{2}_{0} / 2\hbar ^3\omega _{0}}$$

In particular, for $\gamma = 0$ we get: $g_c\approx 0.87(\hbar / ma^{2}_{0}\omega _{0})^{1/4}$. Hence, for the overwhelming majority of various systems $g_c \le 10$.

Notice that an application of an external magnetic field will cause the decay of singlet bipolarons considered here since the energy of an individual polaron in a magnetic field *H* shifts by $-g_L \mu _B H/2$, where $g_L$ is Lande factor, $\mu _B = |e|\hbar /2mc$ is a Bohr magneton. Being singlet, bipolarons do not experience such a shift. Hence, the region of a bipolaron stability is determined by the inequality $H < H_c$, where:

$$H_c = \frac{1}{\sqrt{2\pi }y_{min}(\gamma )}\left( \frac{\gamma _c - \gamma }{\gamma }\right) \frac{ma^{2}_{0}}{\hbar ^2}\frac{g^4}{\hbar ^2\omega ^{2}_{0}}$$

This estimate is valid for the case of non-quantizing magnetic fields.

As is known, the main mechanism leading to finite resistance in solid bodies is dissipation of charge carriers on phonons Ziman (1960). In the case of translation invariant bipolarons the separation of the system into bipolarons and optical phonons is pointless. For a translation invariant bipolaron in the strong coupling limit, the wave function of the system cannot be divided into electron and phonon parts. The total momentum of a translation invariant bipolaron is a conserving value, the relevant wave function is delocalized over the space and a translation invariant bipolaron occurring in a system consisting only of electrons and phonons, will be superconducting. Inclusion of acoustical phonons into consideration leads to a limitation on the possible value of the velocity *v* of a translation invariant polaron or bipolaron at which they have superconducting properties, namely, according to the laws of energy and momentum conservation, this velocity should be less than that of sound *s*. For $v > s$, a translation invariant polaron and bipolaron become dissipative.

In a real system containing defects or structural imperfections with attractive potential, these defects and imperfections will always trap polarons and bipolarons with spontaneously broken symmetry. On the contrary translation invariant bipolarons will form a bound state only if the potential well is deep enough. Otherwise, even in an imperfect system, translation invariant bipolarons will be delocalized. Notwithstanding the lack of bound states in the presence of defects, the total momentum of a bipolaron no longer commutates with the Hamiltonian and therefore is not an integral of the system’s motion. In this case a bipolaron will scatter elastically on a defect as a result of which only its momentum will change. This scattering does not lead to an energy loss. In the absence of dissipation the motion of bipolarons will occur without friction and superconductivity in the system will be retained. In the presence of large defects or imperfections possessing a great trapping (scattering) potential, the system under discussion cannot be considered as infinite any longer.

## Conclusions

In this paper we demonstrate that TI-bipolaron mechanism of Bose condensation can support superconductivity even for infinite chain. According to Fig. 6 the condensation in 1D systems is the phase transition of second kind.

The theory resolves the problem of the great value of the bipolaron effective mass. As a consequence, formal limitations on the value of the critical temperature of the transition are eliminated too. The theory quantitatively explains such thermodynamic properties of HTSC-conductors as availability and value of the jump in the heat capacity lacking in the theory of Bose condensation of an ideal gas. The theory also gives an insight into the occurrence of a great ratio between the width of the pseudogap and $T_c$. It accounts for the small value of the correlation length and explains the availability of a gap and a pseudogap in HTSC materials.

Accordingly, isotopic effect automatically follows from expression (43), where the phonon frequency $\omega _0$ acts as a gap.

Earlier the 3D TI-bipolaron theory was developed by author in Lakhno (2010, 2012, 2013, 2015b). Consideration of 1D case carried out in the paper can be used to explain 3D high-temperature superconductors (3D TI-bipolaron theory of superconductivity was developed in Lakhno 2015a) where 1D stripes play a great role. As the consideration suggests, artificially created nanostripes with enhanced concentration of charge carriers can be used to increase the critical temperature of superconductors. Theoretical description of the nanostripes can also be based on the approach developed.

## References

[CR1] Altmore F, Chang AM (2013). One dimensional superconductivity in nanowires.

[CR2] Basko DM, Conwell EM (2002). Effect of solvation on hole motion in DNA. Phys Rev Lett.

[CR3] Benneman KH, Ketterson JB (2008). Superconductivity: conventional and unconventional superconductors 1–2.

[CR4] Conwell EM, Rakhmanova SV (2000). Polarons in DNA. PNAS.

[CR5] Devreese JT, Peeters F (1984). Polarons and excitons in polar semiconductors and ionic crystals.

[CR6] Devreese JT, Alexandrov AS (2009). Fröhlich polaron and bipolaron: recent developments. Rep Prog Phys.

[CR7] Emin D (1986). Self-trapping in quasi-one-dimensional solids. Phys Rev B.

[CR8] Emin D, Ye J, Beckel CL (1992). Electron-correlation effects in one-dimensional large-bipolaron formation. Phys Rev B.

[CR9] Emin D (2013). Polarons.

[CR10] Fialko NS, Lakhno VD (2000). Nonlinear dynamics of excitations in DNA. Phys Lett A.

[CR11] Firsov YA (1975). Polarons.

[CR12] Ginzburg VL (1968). Problema vysokotemperaturnoy sverhprovodimosti. UFN.

[CR13] Gunnarsson O, Rösch O (2008). Interplay between electronphonon and Coulomb interactions in cuprates. J Phys Condens Matter.

[CR14] Heisenberg W (1930). Die Selbstenergie des Elektrons. ZS F Phys.

[CR15] Holstein T (1959). Studies of polaron motion: part I. The molecular–crystal model. Ann Phys.

[CR16] Hubbard J (1963). Electron correlations in narrow energy bands. Proc R Soc Lond A.

[CR17] Inzelt G (2008). Conducting polymers.

[CR18] Ishiguro T, Yamaji K, Saito G (1998). Organic superconductors.

[CR19] Kashirina NI, Lakhno VD (2010). Large-radius bipolaron and the polaron–polaron interaction. Phys Usp.

[CR20] Kashirina NI, Lakhno VD (2013). Mathematical modeling of autolocalized states in condensed media.

[CR21] Kashirina NI, Lakhno VD (2014). Continuum model of the one-dimensional Holstein bipolaron in DNA. Math Biol Bioinform.

[CR22] Kashirina NI, Lakhno VD (2015). Bipolaron in anisotropic crystals (arbitrary coupling). Math Biol Bioinform.

[CR23] Kashirina NI, Lakhno VD, Tulub AV (2012). The virial theorem and the ground state problem in polaron theory. JETP.

[CR24] Korepin VE, Eßler FHL (1994). Exactly solvable models of strongly correlated electrons. Advanced series in mathematical physics.

[CR25] Kuper CG, Whitfield GD (1963). Polarons and excitons.

[CR26] Lakhno VD (1994). Polarons and applications.

[CR27] Lakhno VD, Starikov EB, Lewis JP, Tanaka S (2006). Modern methods for theoretical physical chemistry of biopolymers.

[CR28] Lakhno VD (2008). DNA nanobioelectronics. Int J Quantum Chem.

[CR29] Lakhno VD (2010). Energy and critical ionic-bond parameter of a 3D-large radius bipolaron. JETP.

[CR30] Lakhno VD (2012). Translation-invariant bipolarons and the problem of high temperature superconductivity. Solid State Commun.

[CR31] Lakhno VD (2013). Translation invariant theory of polaron (bipolaron) and the problem of quantizing near the classical solution. JETP.

[CR32] Lakhno VD (2014). Large-radius Holstein polaron and the problem of spontaneous symmetry breaking. Prog Theor Exp Phys.

[CR33] Lakhno VD (2015a) TI-bipolaron theory of superconductivity. arXiv:1510.04527 [cond-mat.supr-con]

[CR34] Lakhno VD (2015). Pekar’s ansatz and the strong coupling problem in polaron theory. Phys Usp.

[CR35] Lakhno VD, Sultanov VB (2011). On the possibility of bipolaronic states in DNA. Biophysics.

[CR36] Lakhno VD, Sultanov VB (2012). Possibility of a (bi)polaron high-temperature superconductivity in Poly A/ Poly T DNA duplexes. J Appl Phys.

[CR37] Landau LD (1933). On the motion of electrons in a crystal lattice. Phys Z Sowjetunion.

[CR38] Lebed AG (ed) (2008) The physics of organic superconductors and conductors. Springer series in materials science, vol 110. Springer, Berlin

[CR39] Lee TD, Low F, Pines D (1953). The motion of slow electrons in a polar crystal. Phys Rev.

[CR40] Moriya T, Ueda K (2000). Spin fluctuations and high temperature superconductivity. Adv Phys.

[CR41] Offenhüsser A, Rinaldi R (2009). Nanobioelectronics for electronics, biology, and medicine.

[CR42] Pekar SI (1963) Research in electron theory of crystals (US AEC Transl. AEC-tr-555). United States Atomic Energy Comission. Division of Technical Information, USA, Department of Commerce, Washington; Translated into German: Untersuchungen über die Electronen theorie der Kristalle. Akademie-Verlag, Berlin (1954); Translated from Russian: Issledovaniya po Elektronnoi Teorii Kristallov. GITTL, Moscow-Lenibgrad (1951)

[CR43] Proville L, Aubry S (1998). Mobile bipolarons in the adiabatic Holstein–Hubbard model in one and two dimensions. Phys D.

[CR44] Schüttler H-B, Holstein T (1986). Dynamics and transport of a large acoustic polaron in one dimension. Ann Phys.

[CR45] Tohyama T (2012). Recent progress in physics of high-temperature superconductors. Jpn J Appl Phys.

[CR46] Toyota N, Land M, Müller J (2007). Low dimensional molecular metals. Springer series in solid-state sciences.

[CR47] Tulub AV (1962). Slow electrons in polar crystals. Sov Phys JETP.

[CR48] Williams JM, Ferraro JR, Torn RJ (1992). Organic superconductors: synthesis, structure, properties and theory.

[CR49] Ziman JM (1960). Electrons and phonons.

